# Association of exposure to synthetic phenols and metal(loid)s with early puberty in Spanish girls: a multicentric case–control study

**DOI:** 10.1007/s00431-026-06919-1

**Published:** 2026-04-24

**Authors:** Alicia Olivas-Martinez, Arancha Escribano, Isolina Riaño-Galán, Meritxell Torrebias, Pablo Olmedo, Fernando Gil, Beatriz Suarez, Andrea Rodríguez-Carrillo, Raquel Corripio, Francesca Castiello, M. Zelmira Bosch, Xavier Herrero, Paula S. Ventura-Wichner, Carmen Freire

**Affiliations:** 1 Instituto de Investigación Biosanitaria de Granada (Ibs.GRANADA), 18012 Granada, Spain; 2 Centro de Investigación Biomédica en Red de Epidemiología y Salud Pública (CIBERESP), 28029 Madrid, Spain; 3 Hospital Universitario Virgen de La Arrixaca, 30120 Murcia, Spain; 4 Hospital Universitario Central de Asturias, 33011 Oviedo, Spain; 5 Instituto de Investigación Sanitaria del Principado de Asturias (ISPA), 33001 Oviedo, Asturias, Spain; 6 Department of Medicine, University of Oviedo, 33006 Oviedo, Asturias, Spain; 7 Complejo Hospitalario de VIC, 08500 Barcelona, Spain; 8 Department of Legal Medicine, Toxicology, and Physical Anthropology, School of Medicine, University of Granada, 18016 Granada, Spain; 9 Department of Analytical Chemistry, School of Sciences, University of Granada, 18071 Granada, Spain; 10 Parc Taulí Hospital Universitari, Institut d’Investigació I Innovació Parc Taulí (I3PT-CERCA), UAB, 08208 Sabadell, Barcelona, Spain; 11 Pedriatric Endocrinology Unit, Department of Pediatrics, Germans Trias I Pujol University Hospital, 08916 Barcelona, Spain; 12 Hospital HM Nens, HM Hospitales, Instituto de Investigación Sanitaria HM Hospitales, 08009 Barcelona, Spain; 13 Hospital Sant Jaume de Calella, 08370 Calella, Barcelona, Spain; 14 Hospital Universitario Arnau de Vilanova, 25198 Lleida, Spain

**Keywords:** Bisphenol A, Synthetic phenols, Metalloestrogens, Early puberty, Premature thelarche, Precocious puberty

## Abstract

The incidence of precocious puberty (PP) and its variants in girls has increased over recent decades. Early-life exposure to endocrine disrupting chemicals (EDCs) may contribute to this trend. The purpose of this study is to investigate the association of exposure to several phenolic EDCs and metal(loid)s, both individually and as a mixture, with the risk of early puberty in Spanish girls. This multicentric case-control study recruited 182 girls aged 4-8 years diagnosed with PP (n = 101) or manifesting any sign of early puberty (premature thelarche: n = 74, other: n = 7) and 128 controls in six Spanish hospitals. Bisphenol, paraben, benzophenone (BzP), and metal(loid) concentrations were quantified in spot urine samples. Adjusted logistic regression and quantile g-computation models were used to assess associations of individual chemicals and their mixture, respectively, with risk of early puberty. Increased bisphenol A (BPA) exposure was associated with higher risk of overall early puberty [OR (95% CI) = 1.44 (1.19 – 1.73) per two-fold increase in urinary concentrations], PP [1.69 (1.26 – 2.27)], and premature thelarche [1.29 (1.05 – 1.58)]. Higher total BzPs (3rd tertile) and zinc (Zn) concentrations were also associated with higher risk of early puberty and PP. The mixture showed a significant effect on the risk of overall early puberty [OR (95% CI) = 1.20 (1.04 –1.38)], primarily driven by BPA.

*Conclusion*: These findings suggest that exposure to BPA and BzPs may increase the risk of early puberty in girls. Longitudinal studies are warranted to confirm these findings.

**Table:** 

**What is Known:** • *Exposure to endocrine disrupting chemicals (EDCs), such as bisphenol A (BPA), may accelerate or delay puberty onset, but evidence remains limited and inconsistent.* • *Toxic metals with estrogenic properties such as cadmium have been poorly studied in relation to puberty timing.*
**What is New:** • *Higher BPA, benzophenones, and Zn levels were associated with greater odds of early puberty, including PP and premature thelarche, in Spanish girls.* • *Mixture analysis indicated a significant combined effect on early puberty risk, mainly driven by BPA.*

**Supplementary Information:**

The online version contains supplementary material available at 10.1007/s00431-026-06919-1.

## Introduction

A trend towards earlier female puberty, including earlier breast development (thelarche) and menarche, has been observed over recent decades [1, 2]. Such trends have become a growing public health concern because earlier puberty in girls has been associated with a higher risk of developing different health conditions during adolescence [3] and later in life [4–6]. Puberty timing can be influenced by genetic, hormonal, and environmental factors [7]. Among the latter, exposure to endocrine disrupting chemicals (EDCs) has been suggested to play a major role in the acceleration or delay of puberty onset [3, 8, 9]. Certain EDCs, such as bisphenols, parabens, and benzophenones, may contribute to the acceleration of puberty in girls by binding to estrogen receptors alpha or beta (ERα, β) [9–12]. Bisphenol A (BPA) is widely used in plastics, resins, food packaging, thermal receipts, and clothing [13]. Evidence of the adverse health effects of BPA led to its prohibition for certain uses in some countries and its replacement with the structural analogs bisphenol F (BPF) and bisphenol S (BPS) [13]. For their part, parabens and benzophenones are used in cosmetics, personal care products, food packaging, and textiles for their preservative and ultraviolet filtration properties [14–16]. In addition, some toxic metals, such as cadmium (Cd), lead (Pb), mercury (Hg), and nickel (Ni), are considered metalloestrogens because they can bind to ERα [17, 18], potentially accelerating female pubertal development, while some essential metals may also produce estrogenic effects [19].

Prenatal and/or postnatal exposure to BPA, other synthetic phenols, and Pb has been associated with higher risk of precocious puberty (PP) and premature thelarche in humans [20–27]. In contrast, some authors associated childhood exposure to BPA, Pb, and Ni with delayed premature thelarche [9] and puberty onset [8, 28], while others found no significant relationship [9, 29]. Given these conflicting results, this study investigated the association of exposure to synthetic phenols and metal(loid)s with the risk of early puberty in girls, evaluating both individual and combined chemical effects to better reflect real-life exposure scenarios [30].

## Material and methods

### Study population

This is a multicentric hospital-based case–control study that recruited 182 girls aged 4–8 years with signs of early puberty (101 with PP, 74 with premature thelarche, 6 with early pubarche and 1 with isolated vaginal bleeding) in 2018–2022 from the Pediatric Endocrinology Departments in six Spanish hospitals: San Cecilio University Hospital (HUSC) in Granada; HM Nens Hospital, VIC University Hospital, and Corporació Universitària Parc Taulí-Hospital in Barcelona; Asturias Central University Hospital (HUCA) in Asturias; and Virgen de la Arrixaca University Hospital (HUVA) in Murcia. During the same period, 128 age- and hospital-matched controls (ratio 1:1) were recruited at well-child check-up sessions in the same hospitals (except for HUVA). Cases were included if they manifested any sign of early pubertal development, *i.e.*, uni- or bilateral thelarche, central or peripheral PP, or early pubarche. Control girls had no sign of early puberty, organic disease associated with PP, hormone-related disorder, central nervous system condition, endogenous sex steroid production disorder, or genetic alteration associated with puberty.

### Urinary synthetic phenols and metal(loid)s assessment

Single first morning void urine samples were collected by participants using polypropylene containers and stored at −80 ºC until delivery to the University of Granada, where they remained frozen until analysis. Bisphenols (BPA, BPS, and BPF), parabens (methyl-(MeP), ethyl-(EtP), propyl-(PrP), and butyl-(BuP) paraben), and benzophenones (BP-1, BP-3, BP-6, BP-8, and 4-OH-BP) were detected and quantified by dispersive liquid–liquid microextraction and ultra-high performance liquid chromatography with tandem mass spectrometry (UHPLC-MS/MS), as previously described [31] with minor modifications, using an Acquity UPLC I-Class UHPLC system (Waters, Milford, MA) and Xevo TQ-XS mass spectrometer (Waters). Metal(oid)s were measured by inductively coupled plasma mass spectrometry (Agilent 8900 triple quadrupole ICP-MS, Agilent Technologies). Detailed analytical procedures, including sample preparation and quality control, are provided in the Supplementary Material. Urine specific gravity was measured by handheld refractometer (National Instrument Company, Inc., Baltimore, MD) to adjust chemical concentrations for urine dilution [32].

### Covariates

Sociodemographic data were obtained from questionnaires completed by the parents or guardians on the girls’ age, area of residence, ethnicity, continent of birth, adopted/non-adopted status, and maternal schooling. The questionnaire also gathered information on the presence/absence of acne, body odor, and menarche. The pubertal development of girls was evaluated by pediatric endocrinologists using the Tanner scale for breast and pubic hair development [33]. Weight and height of participants were measured following standardized protocols, and their body mass index (BMI) was calculated and converted to age-specific z-scores based on World Health Organization growth reference standards for children aged 5–19 years [34] using the “zscorer” package in R [35].

### Statistical analysis

Parametric and non-parametric tests were applied to examine differences in sociodemographic and clinical data between cases and controls. Normality of continuous variables was assessed using the Kolmogorov–Smirnov test. Urinary chemical concentrations below the limit of detection (LOD) were assigned LOD/√2 [36], and concentrations were summarized as detection frequencies (DFs) and medians. In addition, the molar sum of each phenolic family and of all phenols analyzed was calculated based on the molecular weight as described in the Supplementary Material.

Logistic regression models were developed to examine the association of individual chemicals (single-exposure models) with the risk of early puberty (all diagnoses), premature thelarche, and PP. Individual and summed chemical concentrations showed non-normal distributions and were therefore natural log-transformed (ln) before inclusion in regression models for chemicals with a DF > 85%. Further models were developed considering (i) tertiles of concentrations (chemicals with DF > 85%); (ii) three groups, based on the LOD and median value (chemicals with DF of 50–85%); and (iii) two groups, detected and undetected (chemicals with DF < 50%). A directed acyclic graph (DAG) (Fig. S1) was created to select potential confounding variables, which included age, hospital, and maternal schooling [37]. Regression estimates were expressed as the odds ratio (OR) with 95% confidence interval (CI) of manifesting any sign of early puberty/premature thelarche/PP per two-fold increase in urine chemical concentration. Quantile g-computation was employed to assess the mixture effect of chemicals detected in > 85% of the girls, including three synthetic phenols (BPA, MeP, BP-3) and five metal(loid)s (As, Cu, Hg, Ni, Zn), by chemical family and all chemicals combined, on the risk of (i) early puberty (all diagnoses), (ii) premature thelarche, and (iii) PP. Regression estimates were expressed as the OR with 95% CI of any diagnosis/premature thelarche/PP for a quantile increase in the mixture concentration. Quantile g-computation was performed by categorizing the mixture components in quartiles using the “qgcomp” package in R [38] with 1000 bootstraps.

Sensitivity analyses included (i) adjusting models for BMI z-score to assess the influence of weight status and (ii) excluding girls from HUVA (absence of controls). *P* < 0.05 was considered significant. Analyses were performed using SPSS 28 (IBM-SPSS, Armonk, NY) and R 4.4.2 (R Foundation for Statistical Computing Platform, Vienna, Austria).

## Results

Cases were on average 1 year older than controls (7.44 vs. 6.24 years) and were more likely to be overweight or obese (44% vs. 24%) (Table 1). Most of the cases lived in urban areas (53%), while most controls resided in rural areas (48%). Over 90% of the girls were Caucasian, were born in Europe, and were not adopted, and most of their mothers had a university education, with no significant difference between groups. Among cases, 56% were diagnosed with PP and 41% with premature thelarche, and most were classified in breast development Tanner stage II (61% and 95%, respectively) and pubic hair Tanner stage I (54% and 84%, respectively) (Tables S1–S2). All controls were in Tanner stage I for both characters, while acne, body odor, and menarche were uncommon (Table S1).

**Table 1:** Sociodemographic characteristics of the study population (*n* = 310)

Variables	Cases (*n* = 182)		Control (*n* = 128)		*p*-value*
	**Mean or *n***	**SD or %**	**Mean or *n***	**SD or %**	
**Age** (years)	7.44	0.78	6.24	1.28	** < 0.001**
**Hospital**					** < 0.001**
*HUSC*	15	8.2	8	6.3	
*HUVA*	51	28.0	-	-	
*HUCA*	22	12.1	21	16.4	
*Nens*	62	34.1	64	50.0	
*Tauli*	14	7.7	12	9.4	
*Vic*	18	9.9	23	18.0	
**Area of residence**					**0.002**
*Rural*	58	31.9	61	47.7	
*Sub-urban*	27	14.8	24	18.8	
*Urban*	97	53.3	43	33.6	
**Ethnicity**					0.097
*Caucasian*	163	89.6	123	96.1	
*Hispanic*	9	4.9	3	2.3	
*Other*	10	5.5	2	1.6	
**Continent of birth**					
*America*	4	2.2	3	2.4	0.466
*Africa*	1	0.6	1	0.8	
*Asia*	6	3.3	1	0.8	
*Europe*	171	93.9	123	96.0	
**Adopted**					
*Yes*	5	2.7	3	2.3	
**Maternal schooling**					0.478
*Up to primary*	22	12.1	14	10.9	
*Secondary*	76	41.8	46	35.9	
*University*	84	46.2	68	53.1	
**BMI z-score**^**†**^	7.72	16.47	2.20	12.39	** < 0.001**
**Weight status**^**†**^					**0.001**
*Underweight*	19	10.4	15	11.7	
*Normal weight*	82	45.1	82	64.1	
*Overweight/obesity*	81	44.5	31	24.2	

*SD* standard deviation

^*^Mann–Whitney (age, weight, BMI z-score), student’s *t*-test (height), chi-squared (hospital, residence, maternal education and weight status) or Fisher’s *F* test (ethnicity, continent of birth and adopted). Significant* p*-values (*p* < 0.05) are in bold emphases

^**†**^BMI z-score values were classified as underweight (< 2 standard deviation [SD]), normal weight (≤ 1 SD to ≥ 2 SD), or overweight/obesity (> 1 SD)

Phenols with the highest concentrations were MeP (median = 2.58 ng/mL in cases vs*.* 3.10 ng/mL in controls), BPA (1.78 vs*.* 0.60 ng/mL), and BP-3 (1.30 vs*.* 0.48 ng/mL) (Table 2). Cases and controls exhibited significant differences in the concentrations of BPA, ∑BPs, BP-3, and ∑Phenols. Non-essential metal(loid)s with the highest concentrations were As (22.20 vs*.* 27.60 ng/mL) and Ni (2.02 ng/mL in both groups), but significant differences between groups were observed for Cu and Zn (Table 2). Exclusion of the girls from HUVA led to significant differences in BP-8 and Pb between cases and controls (Table S3).

**Table 2:** Urinary concentrations (ng/mL) of synthetic phenols and metal(loid)s

Exposure biomarkers	DF (%)	Median	DF (%)	Median	*p*-value*
Synthetic phenols	**Cases (*n* = 181)**		**Controls (*n* = 126)**		
BPA	99.4	1.78	96.8	0.60	** < 0.001**
BPS	35.4	< LOD (0.1)	36.5	< LOD (0.1)	0.84
BPF	10.5	< LOD (0.1)	11.1	< LOD (0.1)	0.86
ΣBPs	-	2.21	-	1.03	** < 0.001**
MeP	97.8	2.58	100	3.10	0.70
EtP	48.1	< LOD (0.1)	50.0	< LOD (0.1)	0.74
PrP	49.7	< LOD (0.1)	46.8	< LOD (0.1)	0.62
BuP	50.3	0.15	58.7	0.38	0.14
ΣPBs	-	6.54	-	6.85	0.82
BP-1	74.6	0.33	67.5	0.21	0.17
BP-3	92.3	1.30	86.5	0.48	**0.010**
BP-6	20.4	< LOD (0.1)	23.0	< LOD (0.1)	0.59
BP-8	30.9	< LOD (0.1)	23.0	< LOD (0.1)	0.13
4-OH-BP	63.5	0.18	67.5	0.20	0.48
ΣBzPs	-	3.34	-	1.96	**0.004**
ΣPhenols	*-*	21.49	*-*	16.45	0.24
Metal(loid)s	**Cases (*n* = 180)**		**Controls (*n* = 127)**		
As	100	22.23	100	27.66	0.72
Cd	76.1	0.05	80.3	0.04	0.38
Cu	99.4	7.15	98.4	6.02	**0.03**
Hg	91.1	0.32	94.5	0.35	0.41
Mn	38.3	< LOD (0.07)	43.3	< LOD (0.07)	0.38
Ni	96.7	2.02	96.1	2.02	0.42
Pb	75.6	0.26	82.7	0.26	0.13
Zn	100	509.99	100	443.85	**0.006**
Specific gravity (median)	1.024		1.023		**-**

ΣBPs: sum of BPA, BPS, and BPF; ΣPBs: sum of MeP, EtP, PrP, and BuP; ΣBzPs: sum of BP-1, BP-3, BP-6, BP-8, and 4-OH-BP; ΣPhenols: sum of ΣBPs, ΣPBs, and ΣBzPs

*4-OH-BP* 4-hydroxibenzophenone, *As* arsenic, *BP-1* benzophenone-1, *BP-3* benzophenone-3, *BP-6* benzophenone-6, *BP-8* benzophenone-8, *BPA* bisphenol A, *BPF* bisphenol F, *BPS* bisphenol S, *BuP* buthylparaben, *Cd* cadmium, *Cu* copper, *DF* detection frequency, *EtP* ethylparaben, *Hg* mercury, *LOD* limit of detection, *MeP* methylparaben, *Mn* manganese, *Ni* nickel, *Pb* lead, *PrP* propylparaben, *SG* specific gravity, *Zn* zinc

^*^Mann*–*Whitney test for phenols, their sums, and metal(loid)s with DF > 8 5%; Chi-squared test for phenols and metal(loid)s with DF < 85% and categorized into detected and non-detected. Significant *p*-values (*p* < 0.05) are in bold emphases

**Fig. 1:**
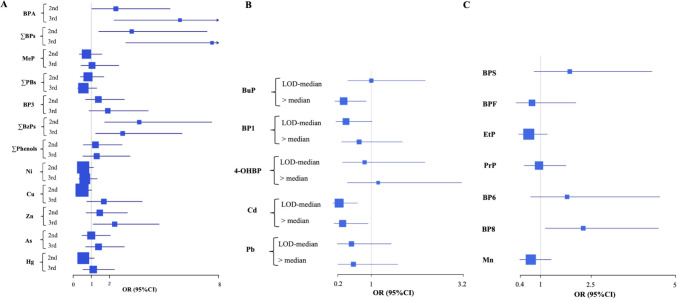
Association between categorized individual synthetic phenols and metal(loid)s and odds of early puberty (all diagnoses). Phenols: 181 cases, 126 controls; metal(loid)s: 180 cases, 127 controls. Models were adjusted for age, hospital, and maternal schooling. **A** Analysis with chemicals detected in > 85% of girls categorized in tertiles. **B** Analysis with chemicals detected in 50–85% of girls categorized into three groups based on the LOD and the median. **C** Analysis with chemicals detected in < 50% of girls categorized into detected and undetected

In adjusted single-exposure models, BPA and ∑BPs were significantly associated with higher risk of early puberty (all diagnoses): OR (95% CI) = 1.44 (1.19–1.73) and 1.62 (1.29–2.04) per two-fold increase in urinary concentrations, respectively (Table 3). In general, results of unadjusted models were like those of adjusted models. In the analysis based on tertiles, girls with BPA and ∑BPs in the 2nd and 3rd vs*.* 1 st tertile showed higher ORs (2.35–5.88 and 3.22–7.64, respectively) (Fig. 1A). In addition, girls with ∑BzPs in the 2nd and 3rd vs*.* 1 st tertile showed a non-monotonic increase in OR (Fig. 1A), while BP-8 detection was also associated with higher risk of early puberty (Fig. 1C). In contrast, BuP concentrations above the median were associated with lower risk of early puberty compared to undetected concentrations (Fig. 1B). Considering cases of PP, BPA and ∑BPs were also associated with higher risk: OR (95% CI) = 1.69 (1.26–2.27) and 1.78 (1.28–2.46), respectively (Table 3). Likewise, BPA and ∑BPs were associated with a higher risk of premature thelarche: OR (95% CI) = 1.29 (1.05–1.58) and 1.47 (1.14–1.90) (Table 3). Additionally, detection of BPS was associated with three-fold higher risk of premature thelarche (95% CI = 1.23–7.68) (data not shown). Among metal(loid)s, Zn was associated with higher risk of early puberty [OR (95% CI) = 1.74 (1.10–2.76)] and PP [2.26 (1.22–4.16)] (Table 3), observing an OR of 2.28 (1.10–4.73) for early puberty in the 3rd vs*.* 1 st tertile (Fig. 1A). In contrast, higher Cd and Cu concentrations were associated with lower risk of early puberty in the analysis of categorized exposures (Fig. 1A–B).

**Table 3:** Association between individual chemicals detected in > 85% of urine samples and odds of early puberty

**Synthetic phenols**		**OR (95% CI)**^**a**^	**OR (95% CI)**^**b**^		**Metal(loid)s**	**OR (95% CI)**^**a**^	**OR (95% CI)**^**b**^
**All diagnoses, *n* = 307 (181 cases, 126 controls)**	**BPA**	1.25 (1.13–1.40)**	1.44 (1.19–1.73)**	***n* = 307 (180 cases, 127 controls)**	**As**	0.99 (0.87–1.12)	1.07 (0.91–1.25)
	**ΣBPs**	1.34 (1.17–1.53)**	1.62 (1.29–2.04)**		**Cu**	1.23 (0.99–1.53)	1.13 (0.84–1.50)
	**MeP**	0.99 (0.91–1.06)	0.98 (0.87–1.10)		**Hg**	0.95 (0.79–1.13)	0.99 (0.79–1.25)
	**ΣPBs**	0.99 (0.89–1.09)	0.93 (0.80–1.07)		**Ni**	0.91 (0.72–1.16)	0.84 (0.62–1.13)
	**BP–3**	1.10 (1.01–1.20)*	1.08 (0.96–1.20)		**Zn**	1.71 (1.20–2.44)*	1.74 (1.10–2.76)*
	**ΣBzPs**	1.13 (1.01–1.27)*	1.12 (0.97–1.30)				
	**ΣPhenols**	1.06 (0.94–1.19)	1.04 (0.87–1.25)				
**Premature thelarche, *n* = 200 (74 cases, 126 controls)**	**BPA**	1.14 (1.01–1.29)*	1.29 (1.05–1.58)*	***n* = 201 (74 cases, 127 controls)**	**As**	1.06 (0.91–1.24)	1.06 (0.87–1.28)
	**ΣBPs**	1.21 (1.03–1.42)*	1.47 (1.14–1.90)*		**Cu**	1.11 (0.85–1.44)	1.02 (0.74–1.41)
	**MeP**	0.96 (0.87–1.05)	0.96 (0.83–1.11)		**Hg**	0.93 (0.74–1.18)	0.97 (0.68–1.21)
	**ΣPBs**	0.96 (0.85–1.08)	0.93 (0.77–1.11)		**Ni**	0.97(0.72–1.30)	0.83 (0.57–1.22)
	**BP–3**	1.05 (0.95–1.17)	1.06 (0.93–1.21)		**Zn**	1.38 (0.90–2.11)	1.39 (0.81–2.38)
	**ΣBzPs**	1.06 (0.92–1.22)	1.09 (0.91–1.30)				
	**ΣPhenols**	0.99 (0.86–1.14)	1.01 (0.81–1.26)				
**PP, *n* = 226 (100 cases, 126 controls)**	**BPA**	1.32 (1.16–1.50)**	1.69 (1.26–2.27)**	***n* = 226 (99 cases, 127 controls)**	**As**	0.92 (0.79–1.07)	1.08 (0.87–1.34)
	**ΣBPs**	1.40 (1.19–1.64)**	1.78 (1.28–2.46)**		**Cu**	1.47 (1.09–1.97)*	1.33 (0.86–2.07)
	**MeP**	0.99 (0.91–1.08)	0.95 (0.82–1.10)		**Hg**	0.94 (0.76–1.15)	1.13 (0.83–1.53)
	**ΣPBs**	0.99 (0.88–1.10)	0.88 (0.73–1.07)		**Ni**	0.92 (0.71–1.20)	0.85 (0.59–1.23)
	**BP–3**	1.13 (1.02–1.25)*	1.09 (0.94–1.27)		**Zn**	1.99 (1.30–3.04)*	**2.26 (1.22–4.16)**
	**ΣBzPs**	1.16 (1.02–1.32)*	1.20 (0.98–1.46)				
	**ΣPhenols**	1.09 (0.95–1.24)	1.00 (0.79–1.28)				

ΣBPs: sum of BPA, BPS, and BPF; ΣPBs: sum of MeP, EtP, PrP and BuP; ΣBzPs: sum of BP-1, BP-3, BP-6, BP-8, and 4-OH-BP; ΣPhenols: sum of ΣBPs, ΣPBs, and ΣBzPs

*95% CI* 95% confidence interval, *OR* odds ratio, *As* arsenic, *BP-3* benzophenone-3, *BPA* bisphenol A, *Cu* copper, *Hg* mercury, *MeP* methylparaben, *Ni* nickel, *Zn* zinc

^*^p-value < 0.05; **p-value < 0.001

^a^Unadjusted OR

^b^Adjusted for age, hospital, and maternal education. Significant associations (*p* < 0.05) are in bold emphases

**Fig. 2:**
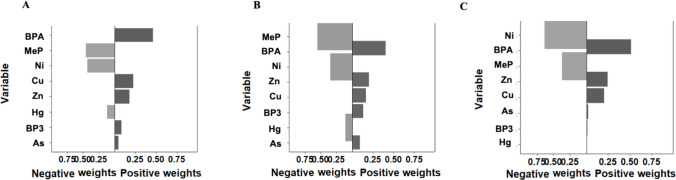
Mixture effect of three synthetic phenols and five metal(loid)s on the odds at early puberty: **A** all diagnoses, **B** premature thelarche, and **C** PP. Dark-colored bars refer to chemicals with an effect in the same direction as the overall effect. Gray-colored bars refer to chemicals with an effect in the opposite direction to the overall effect

The global mixture of phenols and metal(loid)s was associated with 1.20-fold higher risk (95% CI = 1.04–1.38) of early puberty (all diagnoses) and 1.28-fold higher risk (95% CI = 1.03–1.59) of PP for a quartile increase in the mixture concentration (Table 4). This effect was mainly driven by BPA (weight: 46% for all diagnoses and 52% for PP) (Fig. 2 and Fig. S2). In the analysis by chemical family, only the phenol mixture was associated with increased risk of early puberty and PP (Table S4), mainly driven by BPA (Fig. S3).

**Table 4:** Mixture effect of three synthetic phenols and five metal(loid)s on the odds of early puberty

Pubertal outcome	Cases/controls	OR^a^	95% CI	*p*-value
All diagnoses	179/125	1.20	1.04–1.38	**0.01**
Premature thelarche	74/125	1.23	0.86–1.75	0.25
PP	98/125	1.28	1.03–1.59	**0.02**

Mixture components: phenols (BPA, BP-3, and MeP) and metal(loid)s (As, Cu, Hg, Ni, and Zn)

*95% CI* 95% confidence interval, *OR* odds of early puberty (all diagnosis), premature thelarche or PP for a quantile increase in the mixture concentration, *PP* precocious puberty

^a^Adjusted for age, hospital, and maternal education.Significant *p*-values (*p* < 0.05) are in bold emphases

### Sensitivity analysis

Results of main models remained unchanged after adjustment for BMI, and exclusion of the HUVA’s girls yielded similar results in both single-exposure and mixture models (Tables S5–S8; Figs. S2 and S4–S6).

## Discussion

To our best knowledge, this is the first study to assess the association of exposure to synthetic phenols and metal(loid)s with early-life puberty risk in Spanish girls. Findings suggest that higher BPA exposure is associated with an increased risk of early puberty, PP, and premature thelarche; higher BzP levels are associated only with early puberty, whereas Zn is associated with an increased risk of early puberty and PP but not premature thelarche. The mixture analysis suggested a significant combined effect on the risk of early puberty and PP, mainly driven by BPA.

Urinary BPA concentrations in the present girls were in the same range as those observed in children from Spain and elsewhere [39, 40]. A consistent decline in urinary BPA levels among children has been documented over the past two decades [41], likely reflecting regulatory limitations on the use of BPA in infant products and food contact materials [42–45]. Nevertheless, as suggested by the present study, current exposure levels may still represent a threat to sensitive populations. In line with the present findings, case–control studies previously reported associations between urinary or serum BPA and early puberty in girls, including premature thelarche [24, 46] and PP [21, 23, 24, 26]. In our mixture model, BPA was the main contributor to the risk of early puberty, consistent with its well-documented in vitro and in vivo estrogenic activity [11, 47]. However, the epidemiological evidence remains controversial, and several studies found no association between BPA exposure and puberty timing in girls [20, 48–55]. These discrepancies may be attributable, at least in part, to differences in sample size since positive associations have been observed more frequently in larger study populations [20, 21, 26].

The widespread replacement of BPA with BPS and BPF in the manufacture of everyday products has prompted research on the health consequences of daily exposure to these analogs [20, 56]. In line with our findings, a previous case–control study associated BPS exposure with PP in girls, whose urinary BPS concentrations were higher than those found in the present girls [20]. These results are biologically plausible, given that BPS exerts estrogenic activity comparable to that of BPA [11]. Another study observed no link between BPS exposure and premature thelarche [56], but the results are not comparable because they assessed prenatal exposure. Regarding BzPs, two cohort studies associated the exposure of girls aged 4–8 years to BP-3 with accelerated [27] and delayed [57] breast development, while others found no significant association [25, 58, 59]. The known estrogenic activity of BP-8 [60], a derivative of BP-3 and common ultraviolet filter in sunscreens and cosmetics [15, 61], may explain the observed association with increased risk of early puberty, although BzPs showed no relevant effect in the mixture model. Regarding parabens, one cohort study described an association between childhood MeP exposure and earlier breast development [25]; however, others found no relationship [25, 27, 57]. No evidence supports the present finding of a link between BuP exposure and a lower risk of early puberty, whereas previous studies confirm the lack of association between childhood PrP exposure and puberty [25, 27, 57]. Overall, parabens and BzPs may both impact female pubertal development, given their estrogenic properties and wide presence in personal care and cosmetic products [61].

Regarding metal(loid)s, urinary concentrations of As, Hg, and Zn were higher than reported in studies of Spanish and German children [62–65]. In contrast, concentrations of Cd, Ni, Pb, Cu, and Mn were generally lower than those observed in Spanish and European children [62–67]. Despite the known reproductive toxicity and estrogenic properties of Pb [68], the present study found no significant association with early-life puberty, in agreement with other cohort studies [69, 70]. Interestingly, in pubertal girls, urinary Pb concentrations have been positively associated with breast density [70] and negatively associated with inhibin B levels [71], a marker reflecting reproductive axis activation and secondary sexual maturation [72, 73]. One previous cross-sectional study reported an association between Pb blood levels and the risk of premature thelarche [22]. This may indicate that blood is a more appropriate matrix for Pb quantification due to its high potential for accumulation in the body and slower excretion rate [74]. The association of Cd with a lower risk of early-life puberty is consistent with other epidemiological findings that girls with higher Cd levels have later puberty [71] and age at menarche [75]. However, this was an unanticipated result, because Cd is a well-known metalloestrogen [19] and would be expected to have the opposite effect. Nevertheless, the evidence for Cd remains controversial, given that other studies showed no association with pubertal development in girls with higher exposure levels than those of this population [69, 70, 76].

Urinary Zn was positively associated with PP but not premature thelarche. Likewise, two studies found no relationship between urinary Zn levels and breast development or breast density in pubertal girls [70, 76]. The association with PP risk may be explained by the essential role played in the female reproductive system by Zn, which participates in the regulation of ovarian function, acts as a cofactor in the synthesis of sex hormones, and modulates the reproductive axis [77, 78]. In the mixture model, the positive association of Zn with PP persisted but should be considered with caution, as this effect has not previously been reported. In addition, Cu is known to exhibit estrogenic properties [79], and one study of pubertal girls reported a positive association between Cu and breast density [70]. However, the inverse association between Cu and the risk of early puberty should also be interpreted cautiously, given the limited scientific evidence available on this relationship.

The study design and the short half-life of synthetic phenols limit the ability to infer causality. Nevertheless, when daily exposure is relatively stable, as suggested by the moderate intraclass correlation coefficient of BPA (ICC ≈ 0.50) [80], a single urine sample may provide a reasonable estimate of long-term exposure trends. At any rate, future studies should incorporate repeated measurements or utilize alternative matrices better suited for assessing cumulative exposure. In addition, urine is not the appropriate biological matrix to assess chronic exposure to Pb and Hg [81], which may lead to the non-differential misclassification of exposure and potentially bias results towards the null. Further limitations are the lack of controls at HUVA, although the exclusion of HUVA cases yielded similar results. In addition, it is not possible to rule out residual confounding due to unmeasured chemicals, because girls may have been exposed to other EDCs not assessed in this study, which could have influenced observed associations. Finally, multiple comparisons may have increased the risk of detecting spurious associations or overlooking true ones. However, the associations observed in this study were consistently confirmed in models based on categorized exposure biomarkers and in mixture models. Despite these study limitations and the modest effect size observed, these results may have substantive public health relevance due to the extensive and continuous exposure to these chemicals.

Study strengths include the assessment of multiple chemicals and their combined effect, the inclusion of various early-life puberty-related diagnoses, a relatively large sample size (*n* = 310), and a good representation of the Spanish population through participants from different geographic regions.

## Conclusion

This study provides evidence of an association between early puberty in girls and their exposure to common EDCs. A wide variety of EDCs and metal(oid)s were detected in urine samples from girls, and some of them, including BPA and BzPs, were associated with a higher risk of early puberty, premature thelarche, and PP. Given the ubiquitous exposure to these chemicals, the present results underscore the need for longitudinal studies with larger sample sizes to fully clarify their potential role in early puberty development and public health strategies to reduce early-life exposure, including regulatory and educational measures.

## Supplementary Information

Below is the link to the electronic supplementary material.

ESM1 (DOCX 1.26 MB)

## Data Availability

Data will be made available upon request.

## Abbreviations

4-OH-BP: 4-Hydroxybenzophenone
As: Arsenic
BMI: Body mass index
BP-1: Benzophenone-1
BP-3: Benzophenone-3
BP-6: Benzophenone-6
BP-8: Benzophenone-8
BPA: Bisphenol A
BPF: Bisphenol F
BPS: Bisphenol S
BPs: Bisphenols
BuP: Butylparaben
BzPs: Benzophenones
Cd: Cadmium
CI: Confidence interval
Cu: Copper
DAG: Directed acyclic graph
DFs: Detection frequencies
DLLME: Dispersive liquid–liquid microextraction
EDCs: Endocrine disrupting chemicals
ERα: Estrogen receptor alpha
ERβ: Estrogen receptor beta
EtP: Ethylparaben
Hg: Mercury
HUCA: Asturias Central University Hospital
HUSC: San Cecilio University Hospital
HUVA: Virgen de la Arrixaca University Hospital
ICC: Intraclass correlation coefficient
ICP-MS: Inductively coupled plasma mass spectrometry
Ln: Natural log-transformed
LOD: Limit of detection
MeP: Methylparaben
Mn: Manganese
Ni: Nickel
OR: Odds ratio
Pb: Lead
PBs: Parabens
PP: Precocious puberty
PrP: Propylparaben
SD: Standard deviation
SG: Specific gravity
UHPLC-MS/MS: Ultra-high performance liquid chromatography with tandem mass spectrometry
UV: Ultraviolet
Zn: Zinc
