# Supplementary material for: Association of exposure to synthetic phenols and metal(loid)s with early puberty in Spanish girls: a multicentric case–control study
Source: Eur J Pediatr. 2026 Apr 24;185(5):295. doi: 10.1007/s00431-026-06919-1 (PMC13106254; doi:10.1007/s00431-026-06919-1)
Supplement: Supplementary file 1 — (DOCX 1.26 MB) [file 431_2026_6919_MOESM1_ESM.docx]

**SUPPLEMENTARY MATERIAL**

**Supplementary Materials and methods**

**Table S1.** Clinical characteristics of the study population (n=310).

**Table S2**. Distribution of cases (premature thelarche, PP, and others) and controls according to the hospital of recruitment and Tanner stages.

**Table S3.** Urinary concentrations (ng/mL) of synthetic phenols and metal(loid)s excluding girls from HUVA.

**Table S4.** G-computation model for the mixture effect on the odds of early puberty by chemical family.

**Table S5.** Association of individual phenols and metal(loid)s (DF>85%) with the odds of early puberty (all diagnoses), premature thelarche, and PP adjusted for BMI z-score.

**Table S6.** G-computation model for the mixture effect of three phenols and five metal(loid)s on the odds of early puberty (all diagnoses), premature thelarche, and PP adjusted for the BMI z-score.

**Table S7.** Association of synthetic phenols and metal(loid)s (DF>85%) with the odds of early puberty (all diagnoses), premature thelarche, and PP, excluding girls from HUVA.

**Table S8.** G-computation model for the mixture effect of three phenols and five metal(loid)s on the odds of early puberty (all diagnoses), premature thelarche, and PP excluding girls from HUVA.

**Figure S1.** Selection of confounders by directed acyclic graph (DAG).

**Figure S2.** G-Computation model for the association of the chemical mixture with the odds of early puberty (A and G), premature thelarche (B and H), and PP (C and I). Additional g-computation analysis was conducted excluding girls from HUVA [early puberty (D and J), premature thelarche (E and K), and PP (F and L)]. Model 2 was adjusted for age, hospital, and maternal schooling, and Model 3 also included the BMI z-score.

**Figure S3.** G-Computation model for the association of the mixture with the odds early puberty (A, D, G and J), premature thelarche (B, E, H and K), and PP (C, F, I and L) by chemical family. Model 2 was adjusted for age, hospital, and maternal schooling, and Model 3 also included the BMI z-score. Dark-colored bars refer to chemicals with an effect in the same direction as the overall effect. Grey-colored bars refer to chemicals with an effect in the opposite direction to the overall effect.

**Figure S4.** G-computation model for the association of the chemical mixture with the odds of early puberty (A and D), premature thelarche (B and E), and PP (C and F) (all girls: A, B and C; excluding girls from HUVA: D, E and F). Models were adjusted for age, hospital, maternal schooling, and BMI z-score. Dark-colored bars refer to chemicals with an effect in the same direction as the overall effect. Grey-colored bars refer to chemicals with an effect in the opposite direction to the overall effect.

**Figure S5.** Association of individual synthetic phenols and metal(loid)s and odds of early puberty excluding girls from HUVA (all diagnoses; phenols: 131 cases, 126 controls; metal(loid)s: 129 cases, 127 controls). Models were adjusted for age, hospital, and maternal schooling. A) Analysis with chemicals detected in >85% of girls categorized in tertiles, B) Analysis with chemicals detected in 50-85% of girls categorized into three groups based on the LOD and the median. C) Analysis with chemicals detected in <50 % of girls categorized into detected and undetected.

**Figure S6.** G-computation model for the mixture effect of three phenols and five metal(loid)s on the odds of A) early puberty (all diagnoses, n= 254; 129 cases and 125 controls), B) premature thelarche (n= 188; 63 cases and 125 controls), and C) PP (n=184; 59 cases and 125 controls) excluding girls from HUVA. Models were adjusted for age, hospital, and maternal schooling. Dark-colored bars refer to chemicals with an effect in the same direction as the overall effect. Grey-colored bars refer to chemicals with an effect in the opposite direction to the overall effect.

**Supplementary Materials and methods**

**2.2.1. Urinary synthetic phenols and metal(loid)s assessment**

For analysis of synthetic phenols, matrix-matched calibration curves were constructed. The limits of detection (LOD) and quantification (LOQ) were 0.1 and 0.3 ng/mL, respectively, for all studied compounds. Samples were analyzed in two batches that each included standards for calibration curves, 20-25 study samples, blanks, quality control samples, and urine pool controls with mixtures of tested chemicals at low and high concentration levels. The accuracy values for quality control samples were within ±15% of nominal values, and each batch sample was accepted. Finally, recovery rates ranged from 99.1% to 99.7% for spiked urine samples.

For analysis of metal(loid)s, calibration was performed using appropriate standards (Agilent Technologies, Santa Clara, CA, USA) prepared in a solution with 2% (w/v) HNO3 (Suprapur, Merck, Darmstadt, Germany) and 1% (w/v) HCl (Suprapur, Merck) in ultrapure water (Milli-Q, Merck). Urine samples were diluted 1:10 in the above-mentioned solution. Appropriate blanks were analyzed to correct the results. The instrument was tuned, and performance parameters were checked prior to analysis. A solution with 400 µg/L of Sc, Ge, Ir and Rh (ISC Science, Gijón, Spain) was added online as internal standard during analysis to control potential drifts in the signals of the elements tested and possible matrix effects. To ensure the quality of the results, a suitable certified reference material (Seronorm [Sero, Billingstad, Norway] Trace Elements Urine L-1 [reference 210605]) was reanalyzed together with a blank and an intermediate calibration standard every 12 samples. National Institute of Standards and Technology (NIST) (Gaithersburg, MD, USA) Trace Elements in Natural Water Standard Reference Material SRM 1640a was also used as certified reference material and analyzed at the beginning and end of each set of samples. Additionally, one in every 12 samples was reanalyzed at the end of each session. Limits of detection and quantification, respectively, were 0.31 and 0.93 μg/L for As, 0.02 and 0.07 μg/L for Cd, 0.27 and 0.82 μg/L for Cu, 0.08 and 0.24 μg/L for Hg, 0.07 and 0.21 μg/L for Mn, 0.38 and 1.13 μg/L for Ni, 0.06 and 0.18 μg/L for Pb, and 1.26 and 3.77 μg/L for Zn.

**2.4. Statistical Analysis**

The molar sums of bisphenols (∑BPs), parabens (∑PBs), benzophenones (∑BzPs), and total phenols (∑Phenols) were calculated by weighting individual compound concentrations by their molecular weight (Mw), as follows: [∑BPs = (BPA*228.29 + BPS*250.27 + BPF*210.29)/228.29 (Mw_BPA_); ∑PBs = (MeP*152.12 + EtP*166.17 + PrP*180.20 + BuP*194.23)/152.12 (Mw_MeP_); ∑BzPs = (BP1*214.22 + BP3*228.24 + BP6*274.27 + BP8*224.24 + 4-OHBP*198.22)/214.22 (Mw_BP1_); ∑Phenols = ∑BPs + ∑PBs + ∑BzPs].

**Table S1.** Clinical characteristics of the study population (n=310).

| **Variables** | | | **Cases (n=182)** | |  | **Control (n=128)** | |
| --- | --- | --- | --- | --- | --- | --- | --- |
|  |  |  | **n** | **%** |  | **n** | **%** |
| **Clinical diagnostic** | |  |  |  |  |  |  |
|  | *Precocious puberty* |  | 101 | 55.5 |  | - | - |
|  | *Premature thelarche* |  | 74 | 40.7 |  | - | - |
|  | *Others* |  | 7 | 3.8 |  | - | - |
| **Tanner stage** | |  |  |  |  |  |  |
| *Breast development* | |  |  |  |  |  |  |
|  | *B1* |  | 4 | 2.2 |  | 128 | 100.0 |
|  | *B2* |  | 136 | 74.7 |  | - | - |
|  | *B3* |  | 37 | 20.3 |  | - | - |
|  | *B4* |  | 5 | 2.7 |  | - | - |
| *Pubic hair growth* | |  |  |  |  |  |  |
|  | *PH1* |  | 119 | 65.4 |  | 127 | 99.2 |
|  | *PH2* |  | 44 | 24.2 |  | 1 | 0.8 |
|  | *PH3* |  | 15 | 8.2 |  | - | - |
|  | *PH4* |  | 3 | 1.6 |  | - | - |
|  | *PH5* |  | 1 | 0.5 |  | - | - |
| **Other body characters** | |  |  |  |  |  |  |
| **Acne** |  |  |  |  |  |  |  |
|  | *Yes* |  | 14 | 7.7 |  | - | - |
| **Body odor** | |  |  |  |  |  |  |
|  | *Yes* |  | 49 | 26.9 |  | 6 | 4.7 |
| **Menarche** | |  |  |  |  |  |  |
|  | *Yes* |  | 3 | 1.6 |  | - | - |

**Table S2**. Distribution of cases (premature thelarche, PP, and others) and controls according to the hospital of recruitment and Tanner stages.

| **Variables** | | **Cases (n=182)** | | | **Controls (n=128)** |
| --- | --- | --- | --- | --- | --- |
|  |  | **Premature thelarche (n=74)** | **PP**  **(n=101)** | **Others**  **(n=7)** |  |
| **Hospital** | | | | |  |
|  | *HUSC* | 10 | 5 | - | 8 |
|  | *HUVA* | 11 | 40 | - | - |
|  | *HUCA* | 5 | 17 | - | 21 |
|  | *NENS* | 24 | 32 | 6 | 64 |
|  | *TAULI* | 8 | 5 | 1 | 12 |
|  | *VIC* | 16 | 2 | - | 23 |
| **Tanner stage** | | | | |  |
| *Breast development* | | |  |  |  |
|  | *B1* | *-* | 1 | 3 | 128 |
|  | *B2* | *70* | 62 | 4 | - |
|  | *B3* | *3* | 34 | - | - |
|  | *B4* | *1* | 4 | - | - |
| *Pubic hair growth* | | |  |  |  |
|  | *PH1* | *62* | 55 | 2 | 127 |
|  | *PH2* | *9* | 31 | 4 | 1 |
|  | *PH3* | *-* | 11 | 1 | - |
|  | *PH4* | *3* | 4 | - | - |
|  | *PH5* | *-* | 1 | - | - |

**Table S3.** Urinary concentrations (ng/mL) of synthetic phenols and metal(loid)s excluding girls from HUVA.

|  | **DF (%)** | **Median** | **DF (%)** | **Median** | **p-value*** |
| --- | --- | --- | --- | --- | --- |
| **Synthetic**  **Phenols** | **Cases (n=131)** | | **Controls (n=126)** | |  |
| **BPA** | 99.2 | 1.75 | 96.8 | 0.60 | **<0.001** |
| **BPS** | 44.3 | <LOD (0.1) | 36.5 | <LOD (0.1) | 0.20 |
| **BPF** | 8.40 | <LOD (0.1) | 11.1 | <LOD (0.1) | 0.46 |
| **ΣBPs** | - | 2.15 | - | 1.03 | **<0.001** |
| **MeP** | 97.7 | 2.89 | 100 | 3.10 | 0.92 |
| **EtP** | 45.8 | <LOD (0.1) | 50.0 | <LOD (0.1) | 0.50 |
| **PrP** | 49.6 | <LOD (0.1) | 46.8 | <LOD (0.1) | 0.65 |
| **BuP** | 49.6 | 0.15 | 58.7 | 0.38 | 0.14 |
| **ΣPBs** | - | 6.46 | - | 6.85 | 0.95 |
| **BP-1** | 70.2 | 0.31 | 67.5 | 0.21 | 0.63 |
| **BP-3** | 89.3 | 1.15 | 86.5 | 0.48 | 0.10 |
| **BP-6** | 28.2 | <LOD (0.1) | 23.0 | <LOD (0.1) | 0.34 |
| **BP-8** | 38.9 | <LOD (0.1) | 23.0 | <LOD (0.1) | **0.006** |
| **4-OH-BP** | 72.5 | 0.21 | 67.5 | 0.20 | 0.38 |
| **ΣBzPs** | - | 3.22 | - | 1.96 | **0.01** |
| **ΣPhenols** | *-* | 19.340 | *-* | 16.45 | 0.35 |
| **Metal(loid)s** | **Cases (n=129)** | | **Controls (n=127)** | |  |
| **As** | 100 | 28.36 | 100 | 27.66 | 0.32 |
| **Cd** | 100 | 0.05 | 80.3 | 0.04 | 0.16 |
| **Cu** | 99.2 | 6.62 | 98.4 | 6.02 | 0.18 |
| **Hg** | 92.2 | 0.37 | 94.5 | 0.35 | 0.80 |
| **Mn** | 34.9 | <LOD (0.07) | 43.3 | <LOD (0.07) | 0.17 |
| **Ni** | 96.1 | 2.01 | 96.1 | 2.02 | 0.38 |
| **Pb** | 72.2 | 0.22 | 82.7 | 0.26 | **0.04** |
| **Zn** | 100 | 506.30 | 100 | 443.80 | **0.02** |

*ΣBPs: sum of BPA, BPS and BPF; ΣPBs: sum of MeP, EtP, PrP and BuP; ΣBzPs: sum of BP1, BP3, BP6, BP8, and 4-OH-BP; ΣPhenols: sum of ΣBPs, ΣPBs and ΣBzPs; 4-OH-BP: 4-hydroxibenzophenone; As: arsenic; BP-1: benzophenone-1; BP-3: benzophenone-3; BP-6: benzophenone-6; BP-8: benzophenone-8; BPA: bisphenol A; BPF: bisphenol F; BPS: bisphenol S; BuP: buthylparaben; Cd: cadmium; Cu: copper; DF: detection frequency; EtP: ethylparaben; Hg: mercury; MeP: methylparaben; Mn: manganese; Ni: nickel; Pb: lead; PrP: propylparaben; Zn: zinc. *Mann-Whitney test for phenols, their sums, and metal(loid)s with DF>85%; Chi-squared test for chemicals with DF<85% and categorized into detected and non-detected. Significant p-values (p<0.05) are in bold.*

**Table S4.** G-computation model for the mixture effect on the odds of early puberty by chemical family.

| **Pubertal outcome** | **Cases/**  **controls** |  | **Phenols** | | | **Metal(loid)s** | | |
| --- | --- | --- | --- | --- | --- | --- | --- | --- |
|  |  |  | **OR** | **95% CI** | **p-value** | **OR** | **95% CI** | **p-value** |
| **All diagnoses** | 179/125 | *Model 1* | **1.18** | **1.07-1.31** | **0.001** | 1.25 | 0.89-1.18 | 0.761 |
|  |  | *Model 2* | **1.42** | **1.09-1.84** | **0.009** | 1.03 | 0.92-1.16 | 0.562 |
|  |  | *Model 3* | 1.09 | 0.99-1-19 | 0.060 | 1.04 | 0.93-1.16 | 0.516 |
| **Premature**  **thelarche** | 74/125 | *Model 1* | 1.08 | 0.85-1.36 | 0.538 | 1.06 | 0.80-1.41 | 0.689 |
|  |  | *Model 2* | 1.04 | 0.84-1.28 | 0.727 | 1.08 | 0.83-1.41 | 0.574 |
|  |  | *Model 3* | 1.04 | 0.84-1.28 | 0.723 | 1.07 | 0.83-1.40 | 0.591 |
| **PP** | 98/125 | *Model 1* | **1.34** | **1.12-1.60** | **0.001** | 1.02 | 0.81-1.30 | 0.844 |
|  |  | *Model 2* | 1.16 | 0.99-1.35 | 0.066 | 1.03 | 0.87-1.21 | 0.756 |
|  |  | *Model 3* | **1.17** | **1.00-1.35** | **0.045** | 1.04 | 0.88-1.23 | 0.667 |

*Chemical family: phenols (BPA, BP-3, and MeP) and metal(loid)s (As, Cu, Hg, Ni, and Zn). 95% CI: 95% confidence interval; Model 1: Unadjusted; Model 2: Adjusted for age, hospital, and maternal schooling; Model 3: Adjusted for age, hospital, maternal schooling, and BMI z-score; OR: Odds of early puberty, premature thelarche or PP for a quantile increase in the mixture concentration; PP: precocious puberty.*

**Table S5.** Association of individual phenols and metal(loid)s (DF>85%) with the odds of early puberty (all diagnoses), premature thelarche, and PP adjusted for BMI z-score.

| **Synthetic phenols** | | **OR (95% CI)^a^** | **Metal(loid)s** | | **OR (95% CI)^a^** |
| --- | --- | --- | --- | --- | --- |
| **All diagnoses**  **n=307**  **181 cases, 126 controls** | **BPA** | **1.42 (1.18-1.71)**** | **n= 307**  **180 cases, 127 controls** | **As** | 1.07 (0.91-1.26) |
|  | **ΣBPs** | **1.97 (1.41-2.75)**** |  | **Cu** | 1.13 (0.85-1.52) |
|  | **MeP** | 0.97 (0.87-1.09) |  | **Hg** | 1.00 (0.80-1.27) |
|  | **ΣPBs** | 0.92 (0.79-1.06) |  | **Ni** | 0.85 (0.63-1.15) |
|  | **BP-3** | 1.07 (0.96-1.20) |  | **Zn** | **1.70 (1.08-2.70)*** |
|  | **ΣBzPs** | 1.12 (0.96-1.30) |  |  |  |
|  | **ΣPhenols** | 1.02 (0.85-1.23) |  |  |  |
| **Premature thelarche**  **n=200**  **74 cases, 126 controls** | **BPA** | **1.27 (1.04-1.57)*** | **n= 201**  **74 cases, 127 controls** | **As** | 1.06 (0.87-1.28) |
|  | **ΣBPs** | **1.72 (1.18-2.51)*** |  | **Cu** | 1.01 (0.74-1.40) |
|  | **MeP** | 0.97 (0.84-1.12) |  | **Hg** | 0.92 (0.68-1.23) |
|  | **ΣPBs** | 0.92 (0.76-1.11) |  | **Ni** | 0.83 (0.57-1.22) |
|  | **BP-3** | 1.06 (0.92-1.21) |  | **Zn** | 1.38 (0.80-2.36) |
|  | **ΣBzPs** | 1.08 (0.91-1.29) |  |  |  |
|  | **ΣPhenols** | 1.00 (0.81-1.25) |  |  |  |
| **PP**  **n=226**  **100 cases, 126 controls** | **BPA** | **1.68 (1.25-2.26)**** | **n=226**  **99 cases, 127 controls** | **As** | 1.11 (0.89-1.38) |
|  | **ΣBPs** | **2.25 (1.40-3.61)**** |  | **Cu** | 1.44 (0.92-2.27) |
|  | **MeP** | 0.94 (0.81-1.10) |  | **Hg** | 1.15 (0.84-1.56) |
|  | **ΣPBs** | 0.86 (0.71-1.05) |  | **Ni** | 0.88 (0.61-1.28) |
|  | **BP-3** | 1.10 (0.94-1.27) |  | **Zn** | **2.18 (1.18-4.03)*** |
|  | **ΣBzPs** | 1.20 (0.98-1.47) |  |  |  |
|  | **ΣPhenols** | 0.98 (0.77-1.26) |  |  |  |

*ΣBPs: Sum of BPA, BPS and BPF; ΣPBs: Sum of MeP, EtP, PrP and BuP; ΣBzPs: Sum of BP-1, BP-3, BP-6, BP-8 and 4-OH-BP; ΣPhenols: sum of ΣBPs, ΣPBs and ΣBzPs; 95% CI: 95% confidence interval; As: arsenic; BP-3: benzophenone-3; BPA: bisphenol A; Cu: copper; Hg: mercury; MeP: methylparaben; Ni: nickel; OR: odds ratio; Zn: zinc. ^a^Adjusted for age, hospital, maternal schooling, and BMI z-score; *p-value <0.05; **p-value <0.001*

**Table S6.** G-computation model for the mixture effect of three phenols and five metal(loid)s on the odds of early puberty (all diagnoses), premature thelarche, and PP adjusted for the BMI z-score.

| **Pubertal outcome** | **Cases/controls** | **OR^a^** | **95% CI** | **p-value** |
| --- | --- | --- | --- | --- |
| **All diagnoses** | 179/125 | 1.20 | 1.04-1.38 | 0.01 |
| **Premature thelarche** | 74/125 | 1.23 | 0.86-1.75 | 0.26 |
| **PP** | 98/125 | 1.31 | 1.05-1.62 | 0.01 |

*Mixture components: phenols (BPA, BP-3 and MeP) and metal(loid)s (As, Cu, Hg, Ni, and Zn). 95% CI: 95% confidence interval; OR: Odds of early puberty, premature thelarche or PP for a quantile increase in the mixture concentration; PP: Precocious puberty. ^a^Adjusted for age, hospital, maternal schooling, and BMI z-score.*

**Table S7.** Association of synthetic phenols and metal(loid)s (DF>85%) with the odds of early puberty (all diagnoses), premature thelarche, and PP, excluding girls from HUVA.

| **Synthetic phenols** | | **OR (95% CI)^a^** | **OR (95% CI)^b^** | **OR (95% CI)**^c^ | **Metal(loid)s** | | **OR (95% CI)^a^** | **OR (95% CI)^b^** | **OR (95% CI)**^c^ |
| --- | --- | --- | --- | --- | --- | --- | --- | --- | --- |
| **All diagnoses**  **n=257**  **131 cases, 126 controls** | **BPA** | **1.21 (1.08-1.35)**** | **1.44 (1.19-1.73)**** | **1.42 (1.18-1.71)**** | **n=256**  **129 cases, 127 controls** | **As** | 1.08 (0.94-1.23) | 1.07 (0.91-1.25) | 1.07 (0.91-1.26) |
|  | **ΣBPs** | **1.31 (1.13-1.51)**** | **1.62 (1.29-2.04)**** | **1.97 (1.41-2.75)**** |  | **Cu** | 1.13 (0.91-1.42) | 1.13 (0.84-1.50) | 1.13 (0.85-1.52) |
|  | **MeP** | 0.99 (0.92-1.08) | 0.98 (0.87-1.10) | 0.97 (0.87-1.09) |  | **Hg** | 0.99 (0.82-1.21) | 0.99 (0.79-1.25) | 1.00 (0.80-1.27) |
|  | **ΣPBs** | 0.99 (0.89-1.10) | 0.93 (0.80-1.07) | 0.92 (0.79-1.06) |  | **Ni** | 0.9 (0.7-1.15) | 0.84 (0.62-1.13) | 0.85 (0.63-1.15) |
|  | **BP3** | 1.07 (0.97-1.17) | 1.08 (0.96-1.20) | 1.07 (0.96-1.20) |  | **Zn** | **1.59 (1.1-2.31)*** | **1.74 (1.10-2.76)*** | **1.70 (1.08-2.70)*** |
|  | **ΣBzPs** | 1.11 (0.98-1.25) | 1.12 (0.97-1.30) | 1.12 (0.96-1.30) |  |  |  |  |  |
|  | **ΣPhenols** | 1.05 (0.93-1.19) | 1.04 (0.87-1.25) | 1.02 (0.85-1.23) |  |  |  |  |  |
| **Premature thelarche**  **n=189**  **63 cases, 126 controls** | **BPA** | 1.12 (0.98-1.28) | **1.29 (1.05-1.58)*** | **1.27 (1.04-1.57)*** | **n= 190**  **63 cases, 127 controls** | **As** | 1.09 (0.92-1.28) | 1.06 (0.87-1.28) | 1.06 (0.87-1.28) |
|  | **ΣBPs** | **1.19 (1.01-1.41)*** | **1.47 (1.14-1.90)*** | **1.72 (1.18-2.51)*** |  | **Cu** | 1.07 (0.82-1.41) | 1.02 (0.74-1.41) | 1.01 (0.74-1.40) |
|  | **MeP** | 0.95 (0.86-1.05) | 0.96 (0.83-1.11) | 0.97 (0.84-1.12) |  | **Hg** | 0.96 (0.75-1.22) | 0.91 (0.68-1.21) | 0.92 (0.68-1.23) |
|  | **ΣPBs** | 0.95 (0.83-1.09) | 0.93 (0.77-1.11) | 0.92 (0.76-1.11) |  | **Ni** | 0.94 (0.70-1.28) | 0.83 (0.57-1.22) | 0.83 (0.57-1.22) |
|  | **BP3** | 1.04 (0.94-1.17) | 1.06 (0.93-1.21) | 1.06 (0.92-1.21) |  | **Zn** | 1.27 (0.82-1.97) | 1.39 (0.81-2.38) | 1.38 (0.80-2.36) |
|  | **ΣBzPs** | 1.07 (0.92-1.24) | 1.09 (0.91-1.30) | 1.08 (0.91-1.29) |  |  |  |  |  |
|  | **ΣPhenols** | 0.98 (0.84-1.14) | 1.01 (0.81-1.26) | 1.00 (0.81-1.25) |  |  |  |  |  |
| **PP**  **n=187**  **61 cases, 126 controls** | **BPA** | **1.27 (1.10-1.46)**** | **1.69 (1.26-2.27)**** | **1.68 (1.25-2.26)**** | **n= 186**  **59 cases, 127 controls** | **As** | 1.06 (0.89-1.25) | 1.08 (0.87-1.34) | 1.11 (0.89-1.38) |
|  | **ΣBPs** | **1.36 (1.14-1.63)**** | **1.78 (1.28-2.46)**** | **2.25 (1.40-3.61)**** |  | **Cu** | 1.33 (0.96-1.84) | 1.30 (0.86-2.07) | 1.44 (0.92-2.27) |
|  | **MeP** | 1.02 (0.91-1.13) | 0.95 (0.82-1.10) | 0.94 (0.81-1.10) |  | **Hg** | 1.02 (0.79-1.30) | 1.13 (0.83-1.53) | 1.15 (0.84-1.56) |
|  | **ΣPBs** | 1.00 (0.88-1.14) | 0.88 (0.73-1.07) | 0.86 (0.71-1.05) |  | **Ni** | 0.91 (0.68-1.22) | 0.85 (0.59-1.23) | 0.88 (0.61-1.28) |
|  | **BP3** | 1.06 (0.95-1.19) | 1.09 (0.94-1.27) | 1.1 (0.94-1.27) |  | **Zn** | **2.02 (1.23-3.31)*** | **2.26 (1.22-4.16)*** | **2.18 (1.18-4.03)*** |
|  | **ΣBzPs** | 1.11 (0.96-1.28) | 1.2 (0.98-1.46) | 1.2 (0.98-1.47) |  |  |  |  |  |
|  | **ΣPhenols** | 1.08 (0.92-1.25) | 1.00 (0.79-1.28) | 0.98 (0.77-1.26) |  |  |  |  |  |

*ΣBPs: sum of BPA, BPS and BPF; ΣPBs: sum of MeP, EtP, PrP and BuP; ΣBzPs: sum of BP-1, BP-3, BP-6, BP-8, and 4-OH-BP; ΣPhenols: sum of ΣBPs, ΣPBs and ΣBzPs; 95% CI: 95% confidence interval; As: arsenic; BP-3: benzophenone-3; BPA: bisphenol A; Cu: copper; Hg: mercury; MeP: methylparaben; Ni: nickel; OR: odds ratio; Zn: zinc. .^a^Unadjusted OR; ^b^Adjusted for age, hospital, and maternal schooling; ^c^Adjusted for age, hospital, maternal schooling, and BMIz-score *p-value <0.05; **p-value <0.001.*

**Table S8.** G-computation model for the mixture effect of three phenols and five metal(loid)s on the odds of early puberty (all diagnoses), premature thelarche, and PP excluding girls from HUVA.

| **Pubertal outcome** | **Cases/controls** |  | **OR** | **95% CI** | **p-value** |
| --- | --- | --- | --- | --- | --- |
| **All diagnoses** | 129/125 | *Model 1* | 1.50 | 1.24-1.82 | **<0.001** |
|  |  | *Model 2* | 1.34 | 1.10-1.63 | **0.003** |
|  |  | *Model 3* | 1.35 | 1.10-1.64 | **0.003** |
| **Premature thelarche** | 63/125 | *Model 1* | 1.32 | 0.85-3.43 | 0.22 |
|  |  | *Model 2* | 1.26 | 0.84-1.92 | 0.26 |
|  |  | *Model 3* | 1.26 | 0.83-1.92 | 0.28 |
| **PP** | 59/125 | *Model 1* | 2.02 | 1.45-2.79 | **<0.001** |
|  |  | *Model 2* | 1.69 | 1.20-2.39 | **0.002** |
|  |  | *Model 3* | 1.73 | 1.24-2.41 | **0.001** |

*Mixture components: phenols (BPA, BP-3 and MeP) and metal(loid)s (As, Cu, Hg, Ni, and Zn). 95% CI: 95% confidence interval; Model 1: Unadjusted; Model 2: Adjusted for age, hospital, and maternal schooling; Model 3: Adjusted for age, hospital, maternal schooling, and BMI z-score; OR: Odds of early puberty, premature thelarche or PP for a quantile increase in the mixture concentration; PP: precocious puberty.*


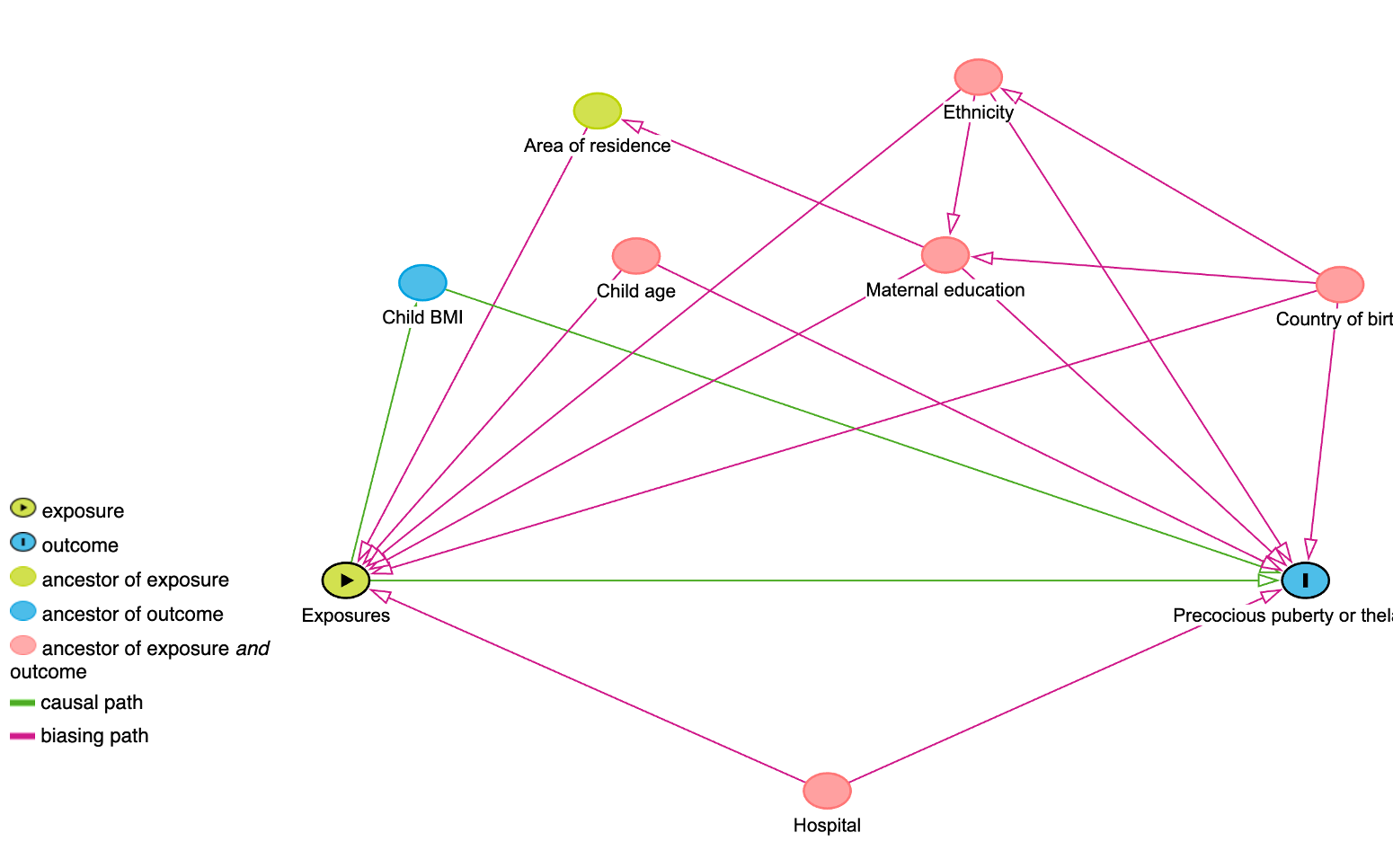
**Figure S1.** Selection of confounders by directed acyclic graph (DAG).


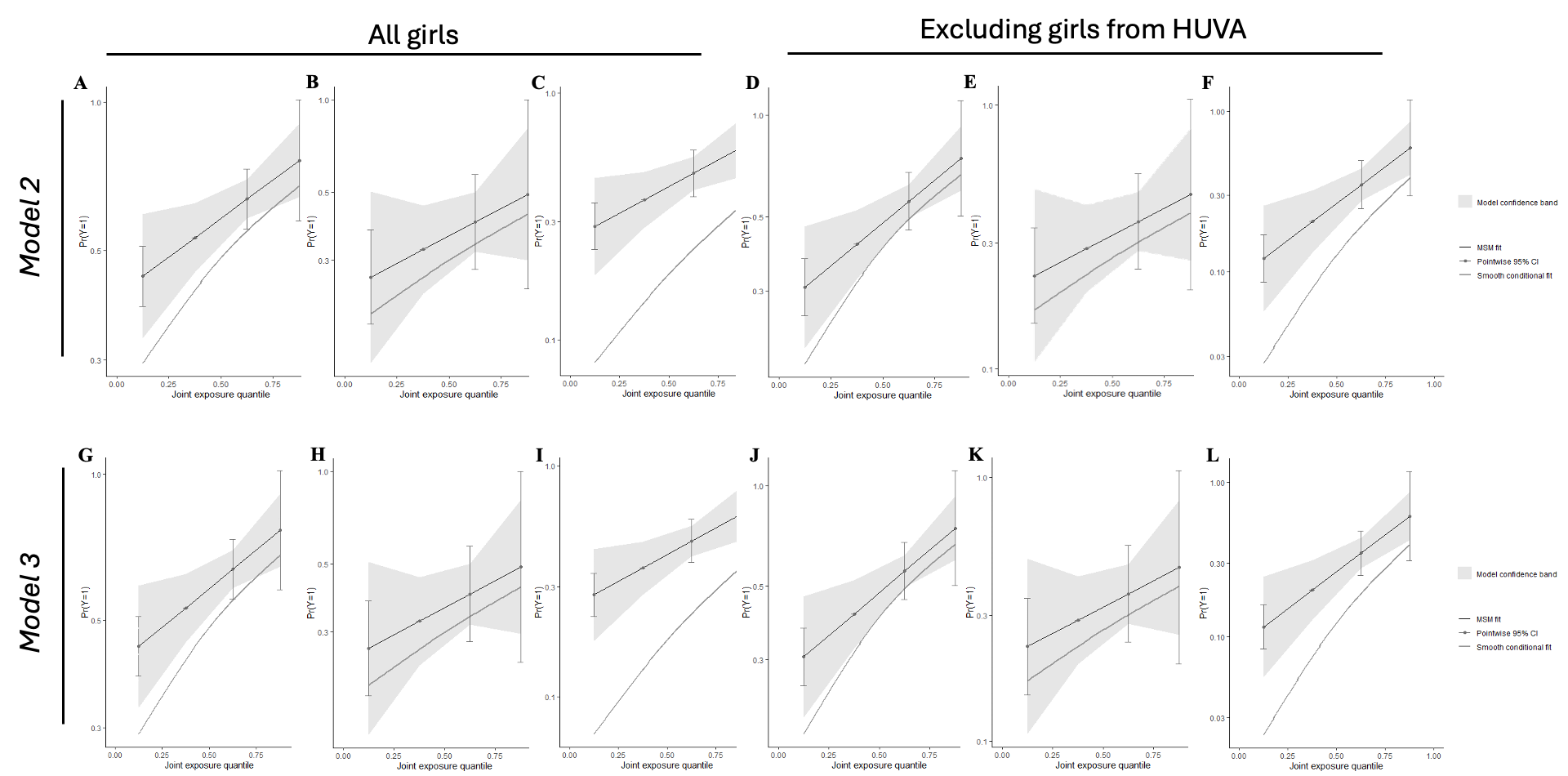


**Figure S2.** G-Computation model for the association of the chemical mixture with the odds of early puberty (A and G), premature thelarche (B and H), and PP (C and I). Additional g-computation analysis was conducted excluding girls from HUVA [early puberty (D and J), premature thelarche (E and K), and PP (F and L)]. Model 2 was adjusted for age, hospital, and maternal schooling, and Model 3 also included the BMI z-score.


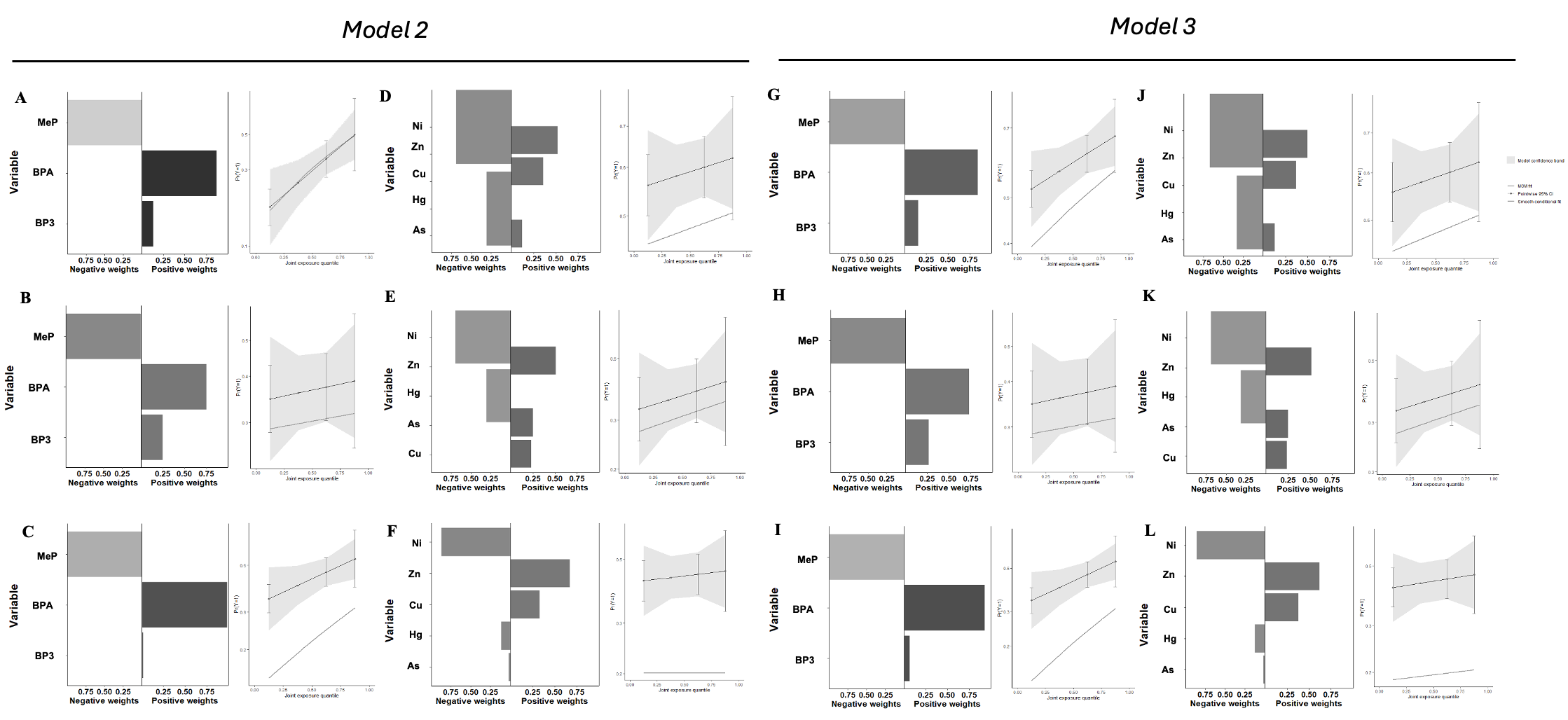


**Figure S3.** G-Computation model for the association of the mixture with the odds early puberty (A, D, G and J), premature thelarche (B, E, H and K), and PP (C, F, I and L) by chemical family. Model 2 was adjusted for age, hospital, and maternal schooling, and Model 3 also included the BMI z-score. Dark-colored bars refer to chemicals with an effect in the same direction as the overall effect. Grey-colored bars refer to chemicals with an effect in the opposite direction to the overall effect.


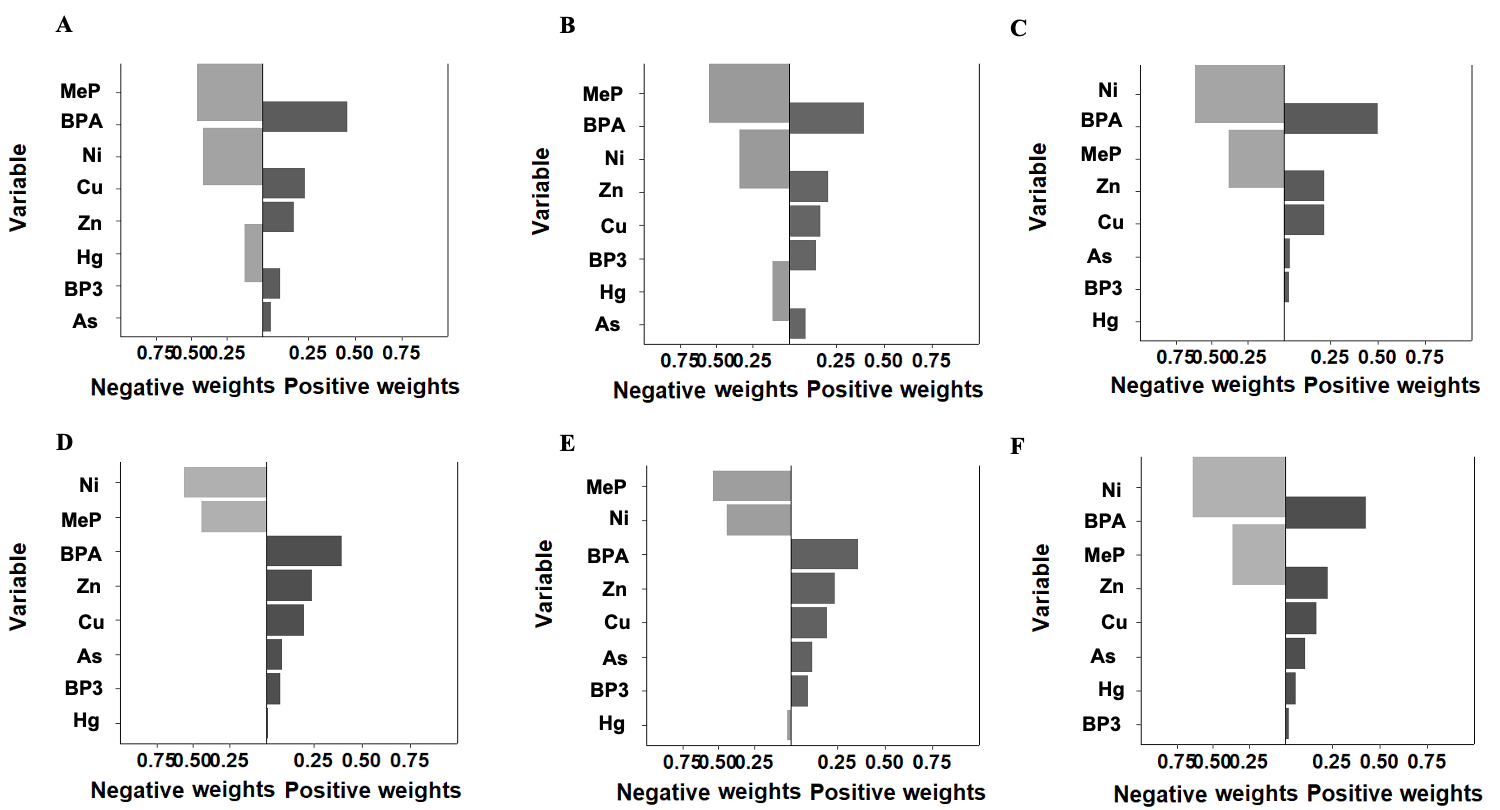


**Figure S4.** G-computation model for the association of the chemical mixture with the odds of early puberty (A and D), premature thelarche (B and E), and PP (C and F) (all girls: A, B and C; excluding girls from HUVA: D, E and F). Models were adjusted for age, hospital, maternal schooling, and BMI z-score. Dark-colored bars refer to chemicals with an effect in the same direction as the overall effect. Grey-colored bars refer to chemicals with an effect in the opposite direction to the overall effect.


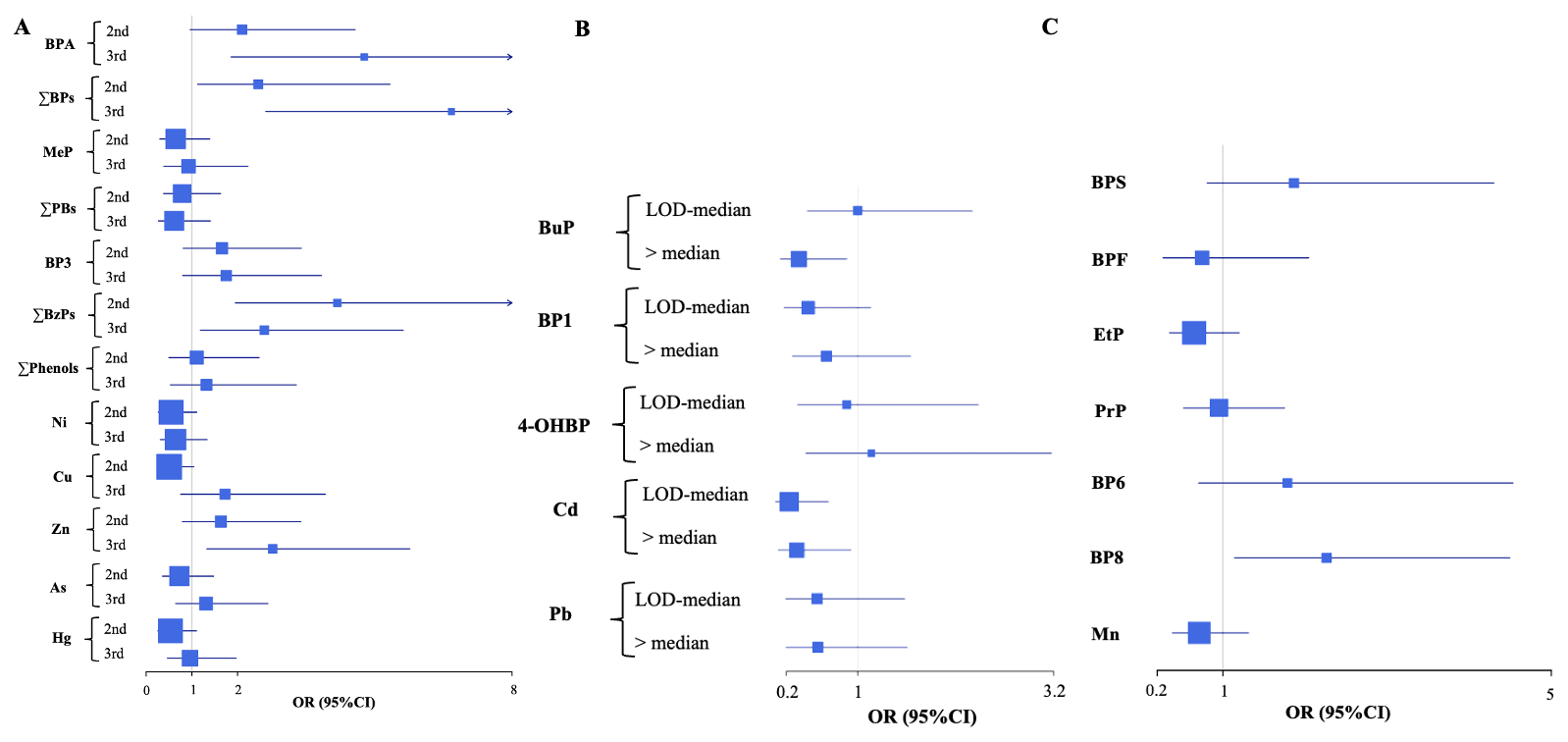
**Figure S5.** Association of individual synthetic phenols and metal(loid)s and odds of early puberty excluding girls from HUVA (all diagnoses; phenols: 131 cases, 126 controls; metal(loid)s: 129 cases, 127 controls). Models were adjusted for age, hospital, and maternal schooling. A) Analysis with chemicals detected in >85% of girls categorized in tertiles, B) Analysis with chemicals detected in 50-85% of girls categorized into three groups based on the LOD and the median. C) Analysis with chemicals detected in <50 % of girls categorized into detected and undetected.


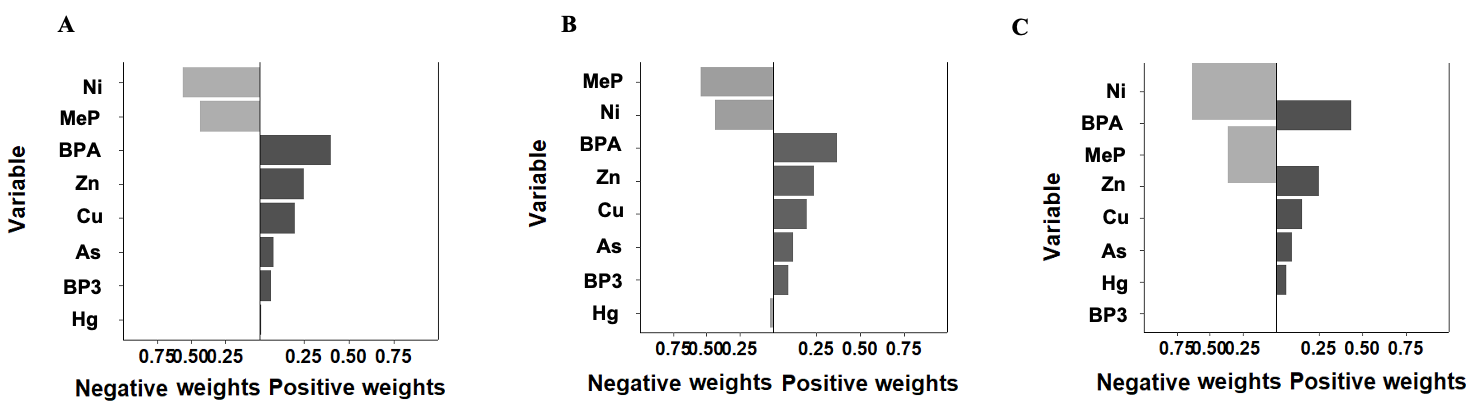


**Figure S6.** G-computation model for the mixture effect of three phenols and five metal(loid)s on the odds of A) early puberty (all diagnoses, n= 254; 129 cases and 125 controls), B) premature thelarche (n= 188; 63 cases and 125 controls), and C) PP (n=184; 59 cases and 125 controls) excluding girls from HUVA. Models were adjusted for age, hospital, and maternal schooling. Dark-colored bars refer to chemicals with an effect in the same direction as the overall effect. Grey-colored bars refer to chemicals with an effect in the opposite direction to the overall effect.
